# Taxonomic position of *Hormaphis similibetulae* Qiao & Zhang, 2004 (Hemiptera, Aphididae): molecular and biological evidences

**DOI:** 10.3897/zookeys.111.1284

**Published:** 2011-06-22

**Authors:** Jing Chen, Li-Yun Jiang, Ge-Xia Qiao

**Affiliations:** 1 Key Laboratory of Zoological Systematics and Evolution, Institute of Zoology, Chinese Academy of Sciences, No. 1 Beichen West Road, Chaoyang District, Beijing 100101, P.R. China; 2 Graduate University of Chinese Academy of Sciences, No. 19 Yuquan Road, Shijingshan District, Beijing 100049, P.R. China

**Keywords:** Hormaphidinae, *Hamamelistes similibetulae*, molecular evidence, biological evidence, new combination, China

## Abstract

The taxonomic position of *Hormaphis similibetulae* Qiao & Zhang, 2004 has been reexamined. The phylogenetic position of *Hormaphis similibetulae* was inferred by maximum parsimony, maximum likelihood and Bayesian analyses on the basis of partial nuclear elongation factor-1α and mitochondrial tRNA leucine/cytochrome oxidase II sequences. The results showed that this species fell into the clade of *Hamamelistes* species, occupying a basal position, and was clearly distinct from other *Hormaphis* species. A closer relationship between *Hormaphis similibetulae* and *Hamamelistes* species was also revealed by life cycle analysis. Therefore, we conclude that *Hormaphis similibetulae* should be transferred to the genus *Hamamelistes* as *Hamamelistes similibetulae* (Qiao & Zhang), **comb. n.**

## Introduction

The aphid tribe Hormaphidini in subfamily Hormaphidinae (Hemiptera: Aphididae) consists of three genera, *Hamamelistes*, *Hormaphis* and *Protohormaphis* (Remaudière and Remaudière 1997). *Hamamelistes* and *Hormaphis* are disjunctively distributed in Eurasia and North America (Guo and Qiao 2005), where they are primarily associated with *Hamamelis* and secondarily associated with *Betula*. The taxonomy of these two genera was once in a mess at both the genus and species levels. They are easily confused with one another, and species of the same genus are difficult to distinguish morphologically. This confusion in the taxonomy was due partly to a limitation of diagnostic characteristics and partly to the fact that no combination had been established between different aphid forms on primary and secondary hosts. Distinction of *Hamamelistes* and *Hormaphis* is based mainly upon alatae, galls and life cycles. However, it is not easy to collect all morphs, and the observation of life cycles takes a long time. Molecular studies have shed light on these issues. Based on the mitochondrial cytochrome oxidase II (COII) gene, Aoki et al. (2001) clarified the Japanese *Hamamelistes* species, established the combination between generations on primary and secondary hosts, and elucidated their life cycles. von Dohlen et al. (2002) estimated the phylogeny of Hormaphidini using partial nuclear elongation factor-1α (EF-1α) and mitochondrial tRNA leucine/cytochrome oxidase II (COII) sequences, the monophyly of both *Hamamelistes* and *Hormaphis* was retrieved with strong support.

Qiao and Zhang (2004) described *Hormaphis similibetulae* based on specimens of apterous viviparous females collected from small conical galls on leaves of *Betula albosinensis* in China (Tibet); the specimens were closely related to *Hormaphis betulae* (Mordvilko) but differed from the latter in body color and living habits. In this study, the taxonomic position of *Hormaphis similibetulae* was reassessed on the basis of nuclear EF-1α and mitochondrial tRNA/COII sequences. A discussion of life cycles was also included.

## Materials and methods

The samples used in this study and the corresponding collection information are listed in Table 1. Eight species of Hormaphidini, covering all the species of *Hamamelistes* and *Hormaphis* were used as ingroups. Three species of Nipponaphidini were chosen as outgroups because Nipponaphidini is considered the sister group of Hormaphidini based on biological and phylogenetic data (Ghosh 1985, von Dohlen and Moran 2000, Ortiz-Rivas and Martínez-Torres 2010). Voucher specimens were preserved in 75% ethanol and deposited in the National Zoological Museum of China, Institute of Zoology, Chinese Academy of Sciences, Beijing, China.

Total genomic DNA was extracted from single aphids preserved in 95% or 100% ethanol using a CTAB protocol modified from Doyle and Doyle (1987). Partial leucine tRNA and the cytochrome oxidase II (COII) gene was amplified with primers 2993+ (Stern 1994) and A3772 (Normark 1996). Sequencing reactions were performed using the corresponding PCR primers from both directions with BigDye Terminator v3.1 Cycle Sequencing Kit (Applied Biosystems, Foster City, CA, USA) and run on an ABI 3730 automated sequencer (Applied Biosystems). Sequences were assembled by Seqman II (DNAStar, Inc., Madison, WI, USA) and verified for protein coding frame-shifts to avoid pseudogenes (Zhang and Hewitt 1996) using Editseq (DNAStar, Inc.). All sequences were deposited in GenBank under the accession numbers JF730745–JF730749. All EF-1α sequences used in this study were downloaded directly from GenBank (for accession numbers see Table 1), and only exons were used for further analysis. Multiple alignments were done with ClustalX 1.83 (Thompson et al. 1997) and then verified manually. Nucleotide composition and pairwise distances based on Kimura’s two-parameter model (K2P) (Kimura 1980) of the aligned sequences were calculated using MEGA 4.0 (Tamura et al. 2007).

Phylogenetic reconstructions were conducted by maximum parsimony (MP), maximum likelihood (ML) and Bayesian analyses for each single gene and a combined dataset. The partition homogeneity test (Farris et al. 1994) based on 100 replicates of a heuristic search algorithm was performed with PAUP*4.0b10 (Swofford 2002) to examine the incongruence between EF-1α and mtDNA. Unweighted MP and ML analyses were carried out using PAUP*. For ML analysis, the best-fit model of nucleotide substitution was selected for each dataset according to the Akaike information criterion (AIC) in Modeltest 3.7 (Posada and Crandall 1998). Heuristic searches were performed with 1000 (MP) or 100 (ML) random-addition sequences and tree-bisection-reconnection (TBR) branch swapping. Bootstrap (BS) analyses were used to assess the relative robustness of branches of the MP (1000 replicates) and the ML (100 replicates) trees (Felsenstein 1985). Bayesian analysis was conducted using MrBayes 3.1.2 (Ronquist and Huelsenbeck 2003) based on the model selected by Modeltest 3.7. In the combined analysis, the mitochondrial and nuclear data were partitioned, and a heterogeneous model was used for each gene partition. The parameters of the model were treated as unknown variables with uniform prior probabilities and were estimated during the analysis. Four Markov chains (three heated and one cold) were run, starting from a random tree and proceeding for one million Markov chain Monte Carlo generations, sampling the chains every 100 generations. Two concurrent runs were conducted to verify the results. The first 2500 trees were discarded as burn-in samples, the remaining trees were used to compute a majority-rule consensus tree with posterior probabilities (PP).

**Table 1.** Collection information and GenBank accession numbers for aphid samples used in this study.

Species	Host	Locality	Date	Voucher	EF-1×	tRNA/COII
*Hamamelistes betulinus* (Horvath)	*Betula davurica*	Japan: Yamanashi, Masutomi	17 Jul. 1998	98081	AF454599*	AF328782*
	*Hamamelis japonica*	Japan: Aomori, Temmabayashi	7 Aug. 1998	98132	AF454596*	AF328775*
	*Betula platyphylla*	Japan: Tokyo, Okutamako	20 May 1999	99121	AF454597*	AF328780*
	*Betula platyphylla*	Japan: Hokkaido, Sapporo	15 Jun. 1999	99187	AF454598*	AF328781*
*Hamamelistes kagamii* (Monzen)	*Hamamelis japonica*	Japan: Yamanashi, Masutomi	17 Jul. 1998	98084	AF454600*	AF328772*
	*Betula grossa*	Japan: Yamanashi, Sanjonoyu	20 May 1999	99118	AF454601*	AF328779*
	*Hamamelis japonica*	Japan: Saitama, Shomaru Pass	8 Jul. 1999	99209	AF454603*	AF328773*
	*Hamamelis japonica*	Japan: Saitama, Shomaru Pass	8 Jul. 1999	99220	AF454602*	AF328774*
*Hamamelistes miyabei*Hamamelistes miyabei (Matsumura)	*Hamamelis japonica*	Japan: Yamanashi, Masutomi	17 Jul. 1998	98086	AF454595*	AF328771*
	*Betula maximowicziana*	Japan: Hokkaido, Sapporo	5 Sep. 1998	98151	AF454593*	AF328776*
	*Betula maximowicziana*	Japan: Gumma, Mt. Akagi	25 May 1999	99146	AF454594*	AF328777*
	*Betula maximowicziana*	Japan: Hokkaido, Sapporo	15 Jun. 1999	99182	AF454592*	AF328778*
*Hamamelistes spinosus* Shimer	*Hamamelis japonica*	USA: Washington, DC	May 1993	93-23	AF454606*	AF328783*
	*Betula nigra*	USA: UT, Logan	28 May 1999	99-54	AF454607*	AF454619*
	*Betula nigra*	USA: WI, Madison	28 Jun. 1999	99-57	AF454608*	None
*Hormaphis betulae* (Mordvilko)	*Betula platyphylla*	Japan: Yamanashi, Masutomi	17 Jul. 1998	98078	AF454609*	None
	*Hamamelis japonica*	Japan: Saitama, Shomaru Pass	21 May 1999	99130	AF454610*	AF454622*
	*Betula platyphylla*	Japan: Tokyo, Kazahari Pass	26 Jul. 1999	99224	AF454611*	AF454623*
	*Betula* sp.	China: Jilin, Ji’an	13 Aug. 2004	15214	DQ493864*	JF730745
*Hormaphis cornu* (Shimer)	*Hamamelis virginiana*	USA: Georgia, Athens	8 Jun. 1994	94-93	AF454612*	AF454621*
*Hormaphis hamamelidis* (Fitch)	*Hamamelis virginiana*	USA: Connecticut, Danielson	1 Aug. 1998	98-05	AF454613*	AF454620*
*Hormaphis similibetulae* Qiao & Zhang	*Betula albosinensis*	China: Tibet, Gongbo’gyamda	5 Jul. 2002	13549	DQ493849*	JF730746
	*Betula albosinensis*	China: Tibet, Linzhi	6 Aug. 2003	15318	DQ493866*	JF730747
*Neohormaphis wuyiensis* Qiao & Jiang	*Quercus* sp.	China: Fujian, Mt. Wuyi	18 Jul. 2003	14525	DQ493858*	JF730748
*Nipponaphis distyliicola* Monzen	*Quercus glauca*	Japan: Shinkiba, Tokyo	16 Apr. 1999	99008	AF454614*	AF454626*
*Thoracaphis quercifoliae* Ghosh	*Quercus* sp.	China: Fujian, Mt. Wuyi	18 Jul. 2003	14526_2	DQ493851*	JF730749

* Sequences from GenBank.

## Results and discussion

The final alignments of EF-1α (excluding three introns) and tRNA/COII sequences consisted of 826 and 761 sites, with 131 and 165 parsimony-informative sites, respectively. A single 1- to 2-base-long indel was found in the tRNA. The genetic distance between two distinct samples of *Hormaphis similibetulae* was 0 for EF-1α and 0.001 for tRNA/COII. The distances of both genes between *Hormaphis similibetulae* and *Hamamelistes* species were much smaller than those between *Hormaphis similibetulae* and the other *Hormaphis* species (EF-1α: average of 0.040 and range of 0.038–0.042 to *Hamamelistes*, average of 0.082 and range of 0.078–0.092 to *Hormaphis*; tRNA/COII: average of 0.080 and range of 0.071–0.085 to *Hamamelistes*, average of 0.106 and range of 0.102–0.112 to *Hormaphis*).

**Figure 1.**
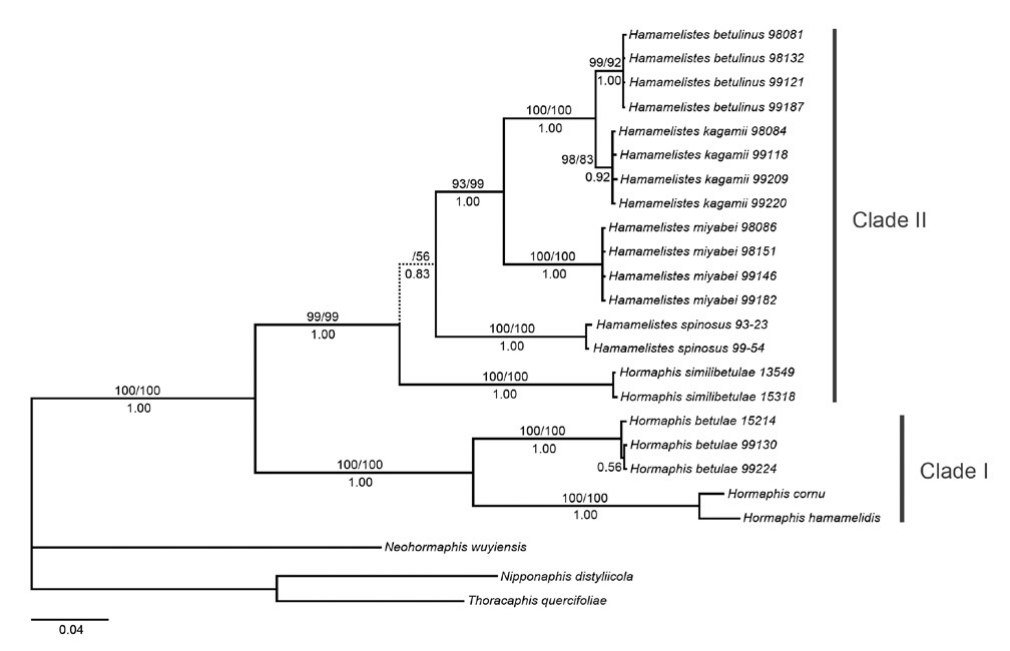
Phylogenetic tree reconstructed from the combined dataset of EF-1α and tRNA/COII sequences. The Bayesian topology and branch lengths are shown. Values above the branches are MP and ML bootstrap percentages, respectively, and Bayesian posterior probabilities are shown below the branches. The broken line indicates inconsistent branch.

For phylogenetic analyses, the partition homogeneity test found no significant conflict between EF-1α and mtDNA (*P*=0.05), indicating that information from both genes could be combined. Combined analysis resulted in similar topology to that obtained in single gene analyses and with higher support for most nodes, so only the combined dataset results were presented. MP analysis yielded eight most parsimonious trees with a length of 611 steps (CI=0.705401, RI=0.845626). ML analysis produced one ML tree based on the optimal model GTR+G selected by AIC in Modeltest 3.7. The 50% majority-rule consensus tree inferred from Bayesian analysis is shown in Fig. 1 and resulted in a topology essentially identical to that obtained in ML analysis, but was different from the strict consensus of MP trees in the position of *Hormaphis similibetulae*. All ingroup taxa constituted a monophyletic group with respect to these outgroups and formed two clades. Clade I (100% MP BS, 100% ML BS, 1.00 PP) was comprised of *Hormaphis betulae*, *Hormaphis cornu*,and *Hormaphis hamamelidis*. Clade II (99% MP BS, 99% ML BS, 1.00 PP) consisted of all the *Hamamelistes* species and *Hormaphis similibetulae*. Within clade II, two distinct samples of *Hormaphis similibetulae* clustered together (100% MP BS, 100% ML BS, 1.00 PP) and were placed as the outermost branch in ML and Bayesian analyses, just as the results based on EF-1α. However, MP analysis revealed the same topology as the mitochondrial analysis: *Hormaphis similibetulae* and *Hamamelistes spinosus* were sister groups, although the support value was low (53% BS), and together formed the basal lineage within clade II.

The results of genetic distances and phylogenetic analyses strongly suggested that *Hormaphis similibetulae* was more closely related to *Hamamelistes* than to *Hormaphis*. *Hormaphis similibetulae* was distinguished by its unique biology, forming galls on leaves of *Betula*. Because of the high morphological similarity with *Hormaphis betulae* (Mordvilko), it was placed under the genus *Hormaphis* (Qiao and Zhang 2004). However, the distinction of apterae of *Hamamelistes* and *Hormaphis* from the secondary host *Betula* is very difficult: both of them are aleyrodiform, dorsoventrally compressed, have body segments fused, short antennae with only 2–4 segments, fore and middle legs without tarsi, and hind legs with rudimentary unsegmented tarsi and lack claws. These reductions appear to be related to the organisms’ sedentary habits on *Betula* and represent the adaptive convergences selected by their temperate habitat. Although species of both genera migrate between *Hamamelis* and *Betula*, their life cycles are quite different and have proven extremely valuable in distinction (Pergande 1901, von Dohlen and Gill 1989, Aoki and Kurosu 1991, von Dohlen and Stoetzel 1991, Aoki et al. 2001). Firstly, *Hamamelistes* have two-year life cycles due to a long gall phase, while *Hormaphis* complete their life cycles within one year. Secondly, on *Hamamelis*, *Hamamelistes* induce spiny or coral-like galls on leaf or flower buds, whereas *Hormaphis* cause conical galls on the leaves. Lastly, *Hamamelistes* inhabit cockscomb-like or blister-like galls on leaves of *Betula*, but *Hormaphis* live freely on the leaves, not causing any deformation. In China, there is only one species of *Hamamelis*, *Hamamelis mollis*, distributed in Sichuan, Hubei, Anhui, Zhejiang, Jiangxi, Hunan and Guangxi Provinces (Zhang and Lu 1995). According to the absence of primary host at high elevations in the Tibetan Plateau, Qiao and Zhang (2004) inferred that *Hormaphis similibetulae* was autoecious on *Betula albosinensis*. We agree with their inference, as *Hamamelis betulinus* and *Hormaphis betulae* were also observed living all year round parthenogenetically on *Betula* in Europe due to lack of primary host (Heie 1980). Although the life cycle of *Hormaphis similibetulae* requires further research, it appears to be more similar to that of *Hamamelistes* than to that of *Hormaphis*.

## Conclusion

The phylogenetic position of *Hormaphis similibetulae* was inferred by MP, ML and Bayesian analyses on the basis of nuclear EF-1α and mitochondrial tRNA/COII sequences. In all phylogenetic analyses, *Hormaphis similibetulae* clustered firmly with *Hamamelistes* and was placed as a basal lineage, clearly differed from other *Hormaphis* species. Life cycle similarities also indicated that *Hormaphis similibetulae* was more closely related to *Hamamelistes* species. We therefore conclude that *Hormaphis similibetulae* should be transferred to the genus *Hamamelistes* as *Hamamelistes similibetulae* (Qiao & Zhang), comb. n.
